# Digital instrument simulator to optimize the development of hyperspectral systems: application for intraoperative functional brain mapping

**DOI:** 10.1117/1.JBO.30.2.023513

**Published:** 2024-12-02

**Authors:** Charly Caredda, Frédéric Lange, Luca Giannoni, Ivan Ezhov, Thiébaud Picart, Jacques Guyotat, Ilias Tachtsidis, Bruno Montcel

**Affiliations:** a Université Claude Bernard Lyon 1, Univ Lyon, INSA-Lyon, UJM-Saint Etienne, CNRS, Inserm, CREATIS UMR, Lyon, France; b University College London, Department of Medical Physics and Biomedical Engineering, London, United Kingdom; c University of Florence, Department of Physics and Astronomy, Firenze, Italy; d Technical University of Munich, München, Germany; e Hospices Civils de Lyon, Service de Neurochirurgie D, Bron, France

**Keywords:** digital simulator, Monte Carlo simulations, intraoperative functional brain mapping, hyperspectral imaging, optical imaging

## Abstract

**Significance:**

Intraoperative optical imaging is a localization technique for the functional areas of the human brain cortex during neurosurgical procedures. These areas can be assessed by monitoring cerebral hemodynamics and metabolism. Robust quantification of these biomarkers is complicated to perform during neurosurgery due to the critical context of the operating room. In actual devices, the inhomogeneities of the optical properties of the exposed brain cortex are poorly taken into consideration, which introduce quantification errors of biomarkers of brain functionality. Moreover, the best choice of spectral configuration is still based on an empirical approach.

**Aim:**

We propose a digital instrument simulator to optimize the development of hyperspectral systems for intraoperative brain mapping studies. This simulator can provide realistic modeling of the cerebral cortex and the identification of the optimal wavelengths to monitor cerebral hemodynamics (oxygenated ${\mathrm{HbO}}_{2}$ and deoxygenated hemoglobin Hb) and metabolism (oxidized state of cytochromes b and c and cytochrome-c-oxidase oxCytb, oxCytc, and oxCCO).

**Approach:**

The digital instrument simulator is computed with white Monte Carlo simulations of a volume created from a real image of exposed cortex. We developed an optimization procedure based on a genetic algorithm to identify the best wavelength combinations in the visible and near-infrared range to quantify concentration changes in ${\mathrm{HbO}}_{2}$, Hb, oxCCO, and the oxidized state of cytochrome b and c (oxCytb and oxCytc).

**Results:**

The digital instrument allows the modeling of intensity maps collected by a camera sensor as well as images of path length to take into account the inhomogeneities of the optical properties. The optimization procedure helps to identify the best wavelength combination of 18 wavelengths that reduces the quantification errors in ${\mathrm{HbO}}_{2}$, Hb, and oxCCO by 47%, 57%, and 57%, respectively, compared with the gold standard of 121 wavelengths between 780 and 900 nm. The optimization procedure does not help to resolve changes in cytochrome b and c in a significant way but helps to better resolve oxCCO changes.

**Conclusions:**

We proposed a digital instrument simulator to optimize the development of hyperspectral systems for intraoperative brain mapping studies. This digital instrument simulator and this optimization framework could be used to optimize the design of hyperspectral imaging devices.

## 1 Introduction

Non-invasive functional brain mapping is a technique that allows the localization of functional areas of the patient’s brain. This technique is used during brain tumor resection surgery to indicate to the neurosurgeon the cortical tissues that should not be removed to avoid motor, speech, and cognitive impairments. Functional magnetic resonance imaging (fMRI)^1^ is the gold standard for preoperative identification of the patient’s functional brain areas. However, after the patient’s craniotomy, neuronavigation is rendered unreliable when brain shift invalidates the patient-to-image registration and, therefore, does not allow a precise identification of the functional areas.^2^ To avoid localization errors, intraoperative MRI has been suggested,^3^ but it is costly and time-consuming, and above all, intraoperative MRI is available only in a very restricted number of neurosurgical centers.^4^ During neurosurgery, electrical brain stimulation (EBS) is the gold standard for intraoperative functional brain mapping,^5^ but this technique is mainly limited by its low spatial resolution (≈5  mm^6^) and has the potential risk to trigger epileptic seizures. This technique allows robust and reliable detection of brain functions but could be traumatic for the patient. Indeed, when testing cognitive functions such as speech, patients are awake, and EBS provokes transient disturbances in the patient that inhibit the function.^5^

Optical imaging is an excellent complement to EBS as this technique is contactless, non-invasive, and non-ionizing and has a low traumatic impact on the patient. Indeed using optical imaging, the task-based paradigms used to localize the brain functions are similar to those used for fMRI.^7^ The analysis of the light absorbance allows to monitor the brain activity (e.g., motor or sensory tasks) with quantification of chromophores in the brain cortex: the concentration changes in oxy-($\Delta C_{{\mathrm{HbO}}_{2}}$) and deoxygenated hemoglobin ($\Delta C_{\mathrm{Hb}}$)^7^^–^^11^ and cytochrome-c-oxidase ($\Delta C_{\mathrm{oxCCO}}$),^12^^–^^14^ a mitochondrial marker of metabolism. CCO is an enzyme in the mitochondria of neuronal cells and is the terminal electron acceptor in the electron transport chain.^15^ Total CCO concentration changes are slow and not correlated with brain activation. However, during cerebral activity, there are symmetrical variations in its oxidized and reduced states.^14^ Near-infrared spectroscopy (NIRS) studies showed that it is possible to resolve changes in the oxidation state of CCO using broadband spectroscopy procedures.^14^^,^^16^^,^^17^ CCO is not the only enzyme that takes part in the electron transport chain; other chromophores are involved such as cytochrome b and c (Cytb, Cytc). In *in vivo* NIRS studies, oxCCO changes are measured with near-infrared light. To observe and measure other chromophores, visible light is required. However, this spectral range is difficult to use *in vivo* due to the poor penetration of the light in the tissue. During neurosurgery, the cerebral cortex is exposed, making it possible to measure cerebral hemodynamics and metabolism at visible and near-infrared wavelengths.

Robust quantification of hemodynamic and metabolism markers is complicated to perform during neurosurgery due to the critical context of the operating room, which makes the calibration of optical devices more complex. To overcome this issue, tissue-simulating objects are required for the development of medical imaging devices. These so-called “phantoms” may be used to evaluate, optimize, compare, or control imaging systems.^18^ Some phantom recipes based on Intralipid and blood^19^ and cytochrome-contained yeast^20^^,^^21^ have emerged to reach that goal. The purpose of these devices is to evaluate the capacity of the acquisition system to follow the variations of oxygenation of the liquid. However, it is not possible to model hemodynamic responses similar to those that occur in the brain. As these phantoms are based on Intralipid and blood, it is not possible to create heterogeneous phantoms that mimic the vascular network of the brain. For this reason, digital brain phantoms may be especially adapted to model hemodynamic and metabolic changes similar to those occurring during cerebral activity^22^^,^^23^ as well as 3D heterogeneous grey matter volumes.^24^

With wide-field imaging devices implying the use of a camera and homogeneous illumination of the cerebral cortex, the biomarkers of brain hemodynamics and metabolism are quantified with the modified Beer–Lambert law.^7^^,^^13^ This spectroscopic technique is however subject to quantification errors such as crosstalks^14^ and partial volume effect.^25^ The crosstalk is defined as a concentration change of a chromophore that induces a change in another chromophore.^17^ The partial volume effect is wavelength-dependent and refers to the impact of a focal change in the optical properties of a portion of the tissue on the surrounding tissue. To reduce the impact of the partial volume effect, the optical mean path length used in the modified Beer–Lambert law needs to be precisely estimated to consider the inhomogeneities of the optical properties.^9^^,^^24^ In NIRS applications, the concentration of the chromophores is usually resolved with a fitting procedure, which requires a great number of wavelengths.^26^ In *in vivo* applications, broadband spectra in the near-infrared range between 780 and 900 nm are adapted for monitoring changes in ${\mathrm{HbO}}_{2}$, Hb, and oxCCO due to the differences between the molar extinction spectra of ${\mathrm{HbO}}_{2}$, Hb, and the strong peak in the molar extinction spectra of oxCCO, as well as the deep penetration of the light into the tissue and low scattering effects. For acquiring broadband spectra for wide-field applications, hyperspectral imaging techniques need to be used.^27^^,^^28^ These broadband systems require complex instrumentation that implies a compromise in the spatial, spectral, and temporal resolution of the acquisitions. Thus, the number of spectral bands in these systems is often limited by the experimental devices. Hyperspectral devices reconstruct spatially and spectrally resolved images. It generates a large amount of data that could be difficult to process in real time. For these reasons, specific wavelengths have been proposed in the literature to monitor hemodynamic and metabolic changes. Bouchard et al.^29^ used a monochrome camera combined with two-wavelength illumination (470 and 530 nm) to monitor hemodynamic changes in a mouse model during cerebral activity. In the same way, White et al.^30^ used two more wavelengths (470, 530, 590, and 625 nm). Arifler et al.^31^ proposed an optimization procedure to identify a reduced number of wavelengths to monitor ${\mathrm{HbO}}_{2}$, Hb, and oxCCO within the spectral range 780 to 900 nm. Authors showed that a combination of eight wavelengths (i.e., 784, 800, 818, 835, 851, 868, 881, and 894 nm) allows a quantification error of <2% compared with a reference measurement (broadband spectra between 780 and 900 nm). Leadley et al.^32^ investigated the capability of NIRS systems to monitor ${\mathrm{HbO}}_{2}$, Hb, and oxCCO within the spectral range of 700 to 900 nm. The authors used an analytical model based on the diffusion equation to explore the effect of wavelength choice, spectral bandwidth, and uncertainties in extinction coefficient and path length on the quantification of concentration changes. The authors showed that a wrong evaluation of the extinction coefficients leads to a poor estimation of ${\mathrm{HbO}}_{2}$, Hb, and oxCCO. In this specific case, NIRS systems that use fewer than 50 measurement wavelengths may not be capable of accurately measuring ${\mathrm{HbO}}_{2}$, Hb, and oxCCO signals.

In this study, we developed a digital instrument simulator to optimize the development of hyperspectral systems for intraoperative brain mapping studies. This simulator can provide realistic modeling of the cerebral cortex and the identification of the optimal wavelengths to monitor cerebral hemodynamics (oxygenated ${\mathrm{HbO}}_{2}$ and deoxygenated hemoglobin Hb) and metabolism (oxidized state of cytochrome b and c and cytochrome-c-oxidase oxCytb, oxCytc, and oxCCO).

## 2 Material and Methods

### 2.1 Digital Instrument Simulator

**Fig. 1:**
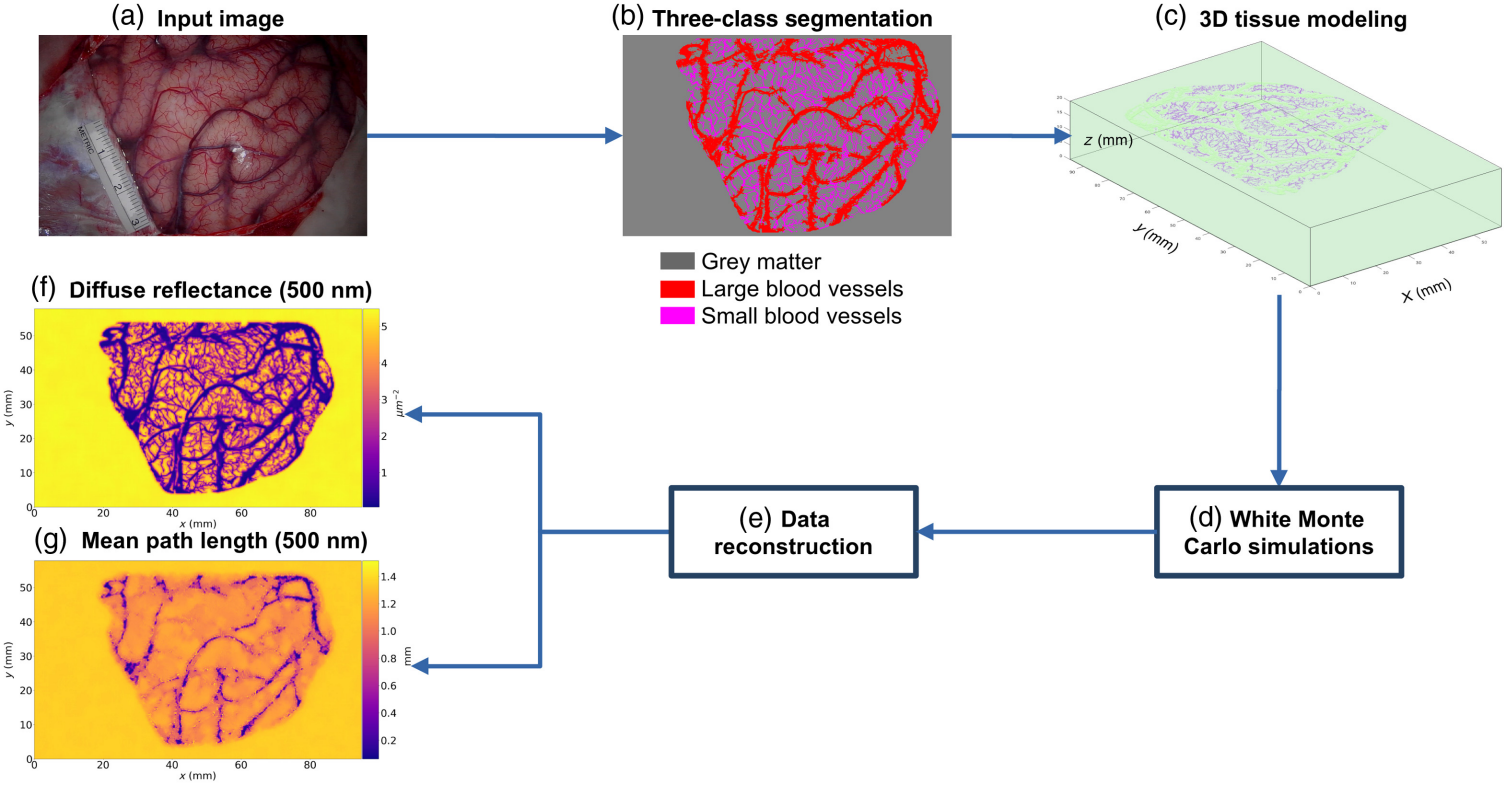
Flowchart of digital instrument simulator. (a) Acquisition of a color image during a neurosurgical operation. (b) Three-class segmentation of the color image (grey matter and large and small blood vessels). (c) 3D tissue modeling. (d) White Monte Carlo simulations of light propagation in the tissue. (e) Reconstruction of diffuse reflectance and mean path length images from white Monte Carlo simulations. (f) Diffuse reflectance image at 500 nm. (g) Mean path length image at 500 nm.

The digital instrument simulator is based on a realistic digital phantom of an exposed cortex computed with Monte Carlo simulations. The code is publicly available on GitHub. This simulator aims to model the light propagation in the exposed brain. It can be used to take into account the inhomogeneities of the optical properties of the exposed brain cortex for functional brain mapping studies and to reduce the quantification errors of biomarkers of brain functionality. The Monte Carlo framework is composed of several steps, see Fig. 1. First, a color image of the exposed cortex was taken during the neurosurgery, see Sec. 2.1.1. This image was segmented into three classes (i.e., grey matter and large and small blood vessels, see Sec. 2.1.2). A brain volume was then modeled, and a white Monte Carlo approach was used to estimate the partial path length, the position, and the angle of exiting packets of photons, see Sec. 2.1.3. Finally, the information of the exiting packets of photons was converted into images of diffuse reflectance and mean path length, see Sec. 2.1.4.

#### 2.1.1 Data collection

A color image of a human brain’s exposed cortex was collected during a brain tumor resection operation at the neurological center of the Pierre Wertheimer Hospital in Bron (France). An 8-bit image was acquired with a surgical microscope Leica M530 OHX, Leica Microsystems SAS, Wetzlar, Germany (1280×720  pixels with a resolution of 73  μm). The study was conducted according to the guidelines of the Declaration of Helsinki and approved by the local ethics committee of Lyon University Hospital (France, Gliospect: 69 HCL14-0270). The participating patients signed written consent.

#### 2.1.2 Image segmentation and brain volume modeling

**Fig. 2:**
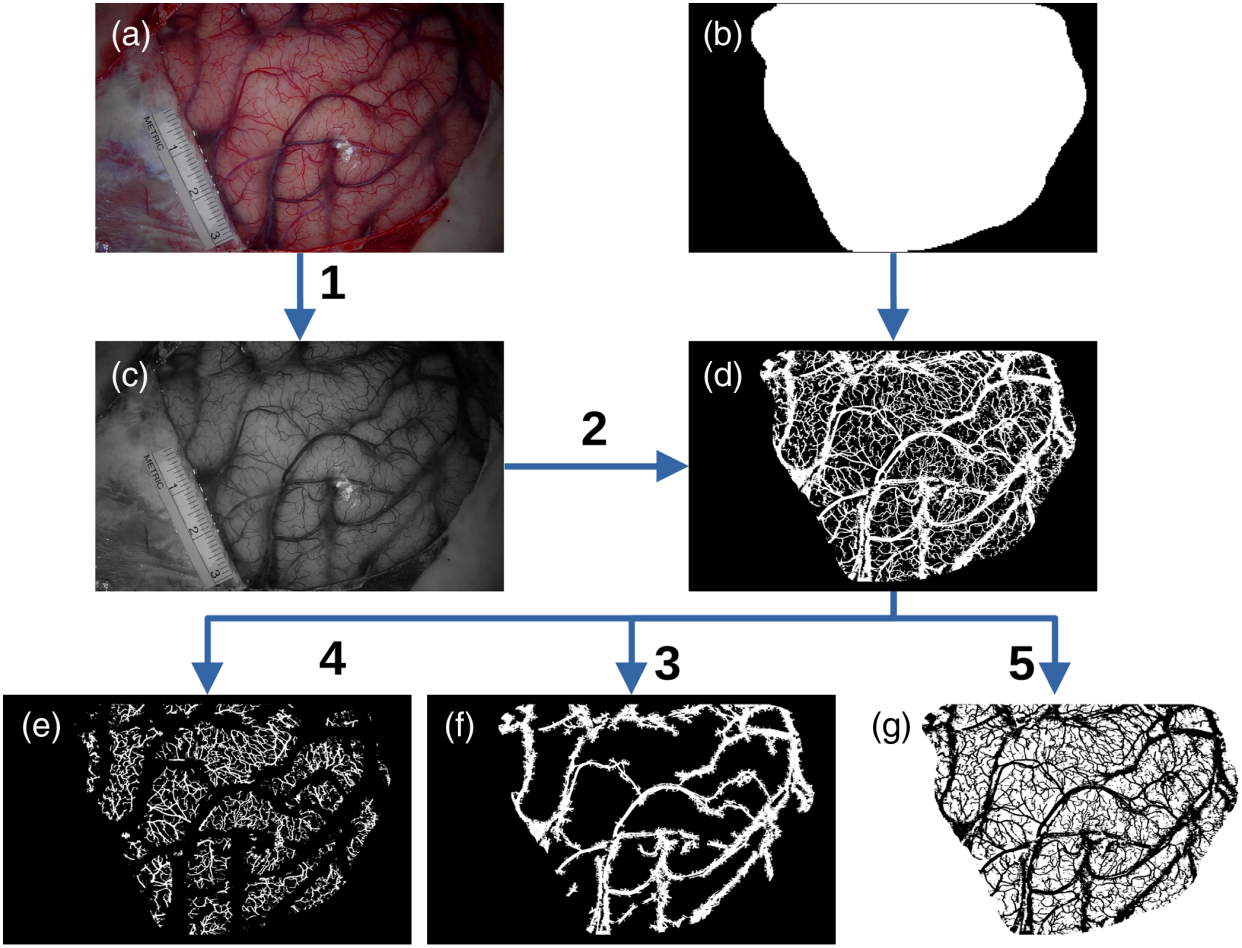
Segmentation of a color image (a) into masks of large blood vessels (f), small blood vessels (e), and grey matter (g). 1: Conversion of color (a) into grayscale image (c). 2: Gaussian adaptive thresholding to obtain the binary image (d). 3: Computation of the large blood vessel mask (f). 4: Computation of the small blood vessel mask (e). 5: Computation of the grey matter mask (g).

The color image was segmented into three classes (i.e., grey matter and large and small blood vessels) using morphological operations, see Fig. 2.

1. The color image (A) was converted into grayscale (C) using the Python library OpenCV (v4.8.1).^33^
2. The grayscale image (C) was converted into a binary image (D) using a Gaussian adaptive threshold with a block size of 5×5  mm. The block size was automatically converted into an odd number of pixels using the resolution of the image (ceil(5/0.073)=69  pixels). A block size of 5 mm corresponded to the optimal size for detecting the blood vessels in the human brain. A smaller or larger size would impact the detection of the large and small blood vessels, respectively. The adaptive threshold was only applied to the exposed cortex by applying the mask (B). The latter is created manually. Saturated pixels due to specular reflection were removed from the image (D) with a simple thresholding of image (C).
3. The small vessels were removed from the binary image (D) using a morphological opening with a disk of 0.5-mm diameter (7 pixels) as a structuring element. Using the resulting image, a morphological closing was computed to get the image (F), the large blood vessel mask.
4. A binary exclusive disjunction (xor) was computed between images (D) and (F) to get the small blood vessel mask (E).
5. The mask of grey matter (G) corresponded to the rest of the pixels.

**Fig. 3:**
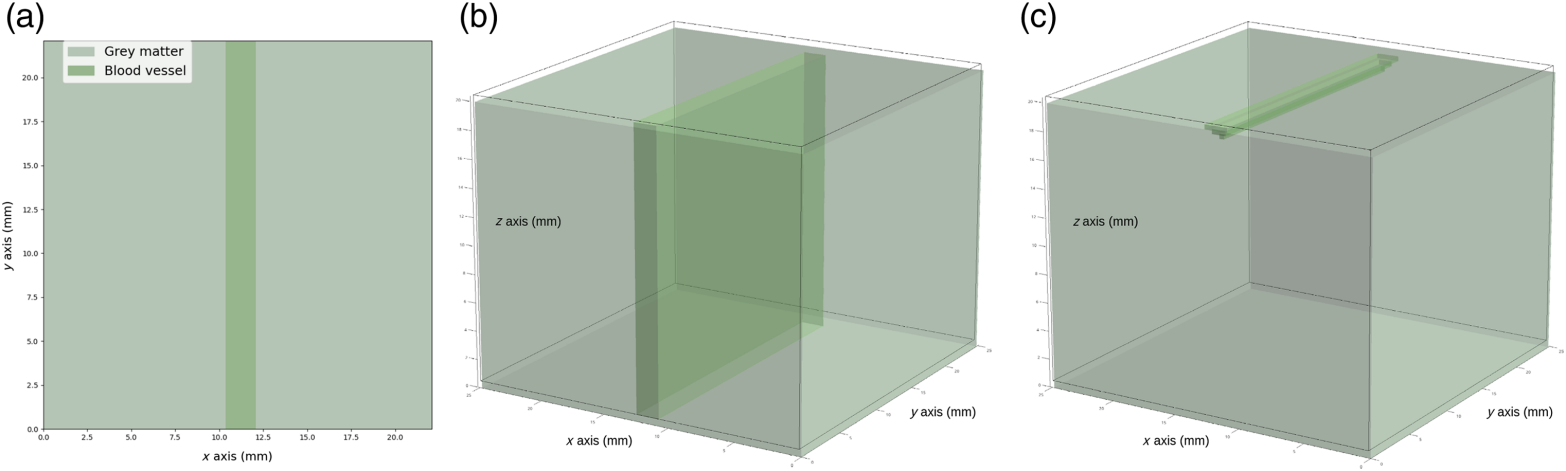
Steps for modeling a brain volume with blood vessels. (a) Segmentation mask of the grey matter and the blood vessels. (b) Replication of the binary segmentation masks along the vertical direction (z axis) on 2 cm. (c) Morphological erosion to create the blood vessel depth.

Once the image was segmented into three classes, we modeled the brain volume. First, the binary segmentation masks were encased in a larger image having the label of grey matter. This operation is performed to prevent a blood vessel label from appearing at the edges of the image. The objective is to avoid photon loss due to the boundary effects during the Monte Carlo simulations, see Sec. 2.1.3. The binary segmentation masks were expanded along the vertical direction (z axis) on 2 cm to convert the binary images into binary volumes, see Figs. 3(a) and 3(b). Then, we modeled the 3D blood vasculature with morphological erosion, see Fig. 3(c). The thickness of the blood vessels was calculated on the basis of the vessel’s diameter. The structuring element used for the erosion was a disk of 0-pixel diameter for z=0 (in pixels) and was increased to 1 pixel while increasing z axis. The binary volumes of the three classes were finally merged together with a final isotropic resolution of 73  μm, see Fig. 1. With this model of the cerebral vasculature, the blood vessels were only modeled on the surface of the volume. Deep blood vessels (which are not visible in color imaging) were not included.

#### 2.1.3 White Monte Carlo simulations

**Fig. 4:**
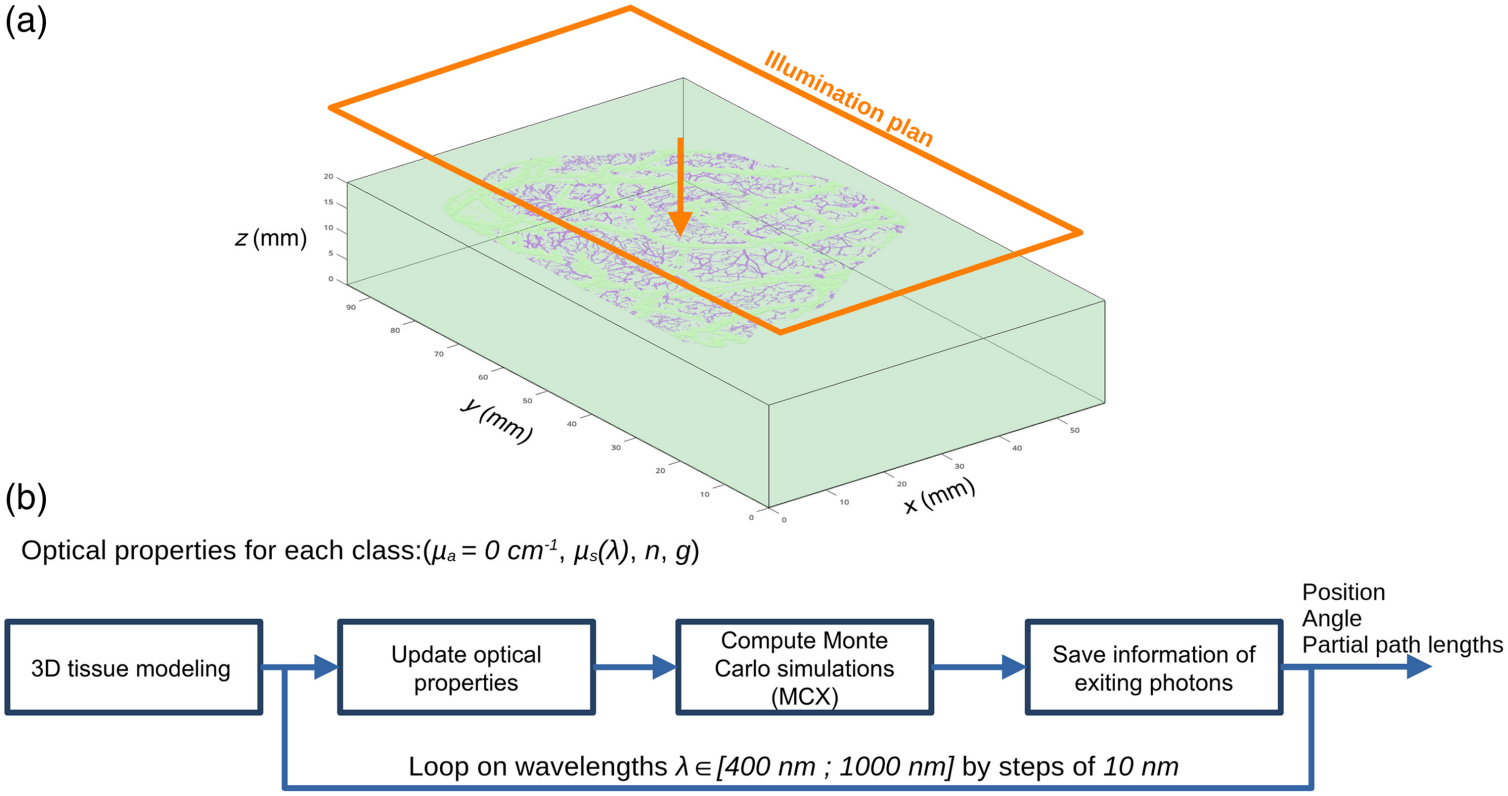
White Monte Carlo simulations. (a) Schematics for the Monte Carlo simulations with Ref. 34. (b) Flowchart of white Monte Carlo simulations.

Once the brain volume was defined, we computed Monte Carlo simulations of light propagation in the domain using MCX^34^ software, see Fig. 4.

A planar light source was placed on the top of the volume to homogeneously illuminate the brain volume. We set the Fresnel reflection as boundary conditions for the top and bottom faces of the volume (i.e., an exiting packet of photons is lost). Cyclic boundary conditions were applied for the sides of the volume to avoid photon loss due to the boundary effects. Each voxel of the volume included optical properties, see Table 1.

**Table 1:** Optical properties used in the white Monte Carlo simulations. λ denotes the wavelength in nanometers.

	Grey matter	Large blood vessels	Small blood vessels
Absorption coefficient $\mu_{a}$ (${\mathrm{mm}}^{-1}$)	0	0	0
Scattering coefficient $\mu_{s}$ (${\mathrm{mm}}^{-1}$)	$4.08{\left(\frac{\lambda }{500}\right)}^{-3.089}$ ^35^	$2.2{\left(\frac{\lambda }{500}\right)}^{-0.66}$ ^35^	$2.2{\left(\frac{\lambda }{500}\right)}^{-0.66}$ ^35^
Anisotropy coefficient g	0.85^36^	0.935^37^	0.935^37^
Refractive index n	1.36^38^	1.4^37^	1.4^37^

For these simulations, we implemented a white Monte Carlo approach, and the absorption coefficient was set to $0\;{\mathrm{mm}}^{-1}$. With this technique, the packets of photons were not affected by the absorption. The advantage of the white Monte Carlo approach is that the absorption can be considered “a posteriori,” using the microscopic Beer–Lambert law.^39^ Thus, when changing the absorption parameters of the volume (i.e., to simulate a brain activation for example), the simulation does not need to be run again, speeding up the processing. In this case, only one simulation per wavelength is required, considering that the scattering properties of the medium do not change. A scattering change can be considered, but a new simulation with a new set of $\mu_{s}$ would need to be rerun. For each wavelength (from 400 to 1000 nm by steps of 10 nm), three outputs were stored for every packet of photons exiting the top face of the volume: the position of the exiting packet of photons (x,y), its exiting angle, and the partial path length (the length that each photon has spent in each class of the domain).

#### 2.1.4 Image reconstruction

Once the white Monte Carlo simulations were performed (see Sec. 2.1.3), a large amount of data were generated for each wavelength (position, angle, and partial path lengths of exiting photons), see Fig. 4. For each wavelength, around 7 Gb of data needs to be processed to reconstruct the reflectance and mean path length images. With the aim of speeding up image reconstruction, a multithreaded program C++ code has been developed. To reconstruct the diffuse reflectance and mean path length images, the absorption coefficients were computed with the chemical composition of each tissue.^35^ These values were taken from the literature and corresponded to a nominal physiological condition,^7^^,^^40^^–^^42^ see Table 2. A cerebral activation will be considered later in the paper, see Sec. 2.2. With our equipment, it is not possible to determine whether the blood vessels identified by the segmentation method are arteries or veins. For this reason, we considered two cases: (1) the large and small blood vessels are arteries, and (2) the large and small blood vessels are veins.

**Table 2:** Chemical composition of the modeled tissue.

	Grey matter	Arteries	Veins
$F_{\text{Water}}$ (%)	73	55	55
$F_{\text{Fat}}$ (%)	10	1	1
$C_{\mathrm{HbT}}$ (μM)	76	2324	2324
${\mathrm{SatO}}_{2}$ (%)	85	98^43^	60^43^
$C_{\mathrm{oxCCO}}$ (μM)	6.4	0	0
$C_{\mathrm{redCCO}}$ (μM)	1.6	0	0
$C_{\mathrm{oxCytb}}$ (μM)	2.37	0	0
$C_{\mathrm{redCytb}}$ (μM)	0.89	0	0
$C_{\mathrm{oxCytc}}$ (μM)	1.36	0	0
$C_{\mathrm{redCytc}}$ (μM)	0.68	0	0

The diffuse reflectance and mean path length images can be reconstructed on a camera sensor that could be located on the tissue surface or outside the volume using a lens system. On the surface of the volume, the number of pixels is at most equal to that of the modeled surface. A spatial binning can be performed to increase the signal-to-noise ratio at the expense of spatial resolution.

To reconstruct the image on the modeled camera sensor using a lens system, we used the transfer-matrix method.^44^ With this approach, we can consider key parameters of the optical system (focal length, working distance, size of the optics, and size of the camera sensor). A lens with a focal length of $f_{0}$ (in mm) was placed at a distance d0 (in mm) from the surface of the tissue (along the axis z), and the sensor was located at a distance s (in mm) from the lens (along the axis z). The optical system is modeled with a system matrix S

$$S=T_{s}L_{f_{0}}T_{d0}=(\begin{array}{cc} 1 & s\\ 0 & 1 \end{array})(\begin{array}{cc} 1 & -\frac{1}{f_{0}}\\ 0 & 1 \end{array})(\begin{array}{cc} 1 & d0\\ 0 & 1 \end{array}),$$ (1)

where $T_{d0}$ and $T_{s}$ denote the translation matrices for the ray in air before and after the lens. $L_{f_{0}}$ is the lens matrix. To model the acquisition of the photon j by the pixel $\left(x_{s},y_{s}\right)$ of the camera sensor, we applied the system matrix S on packets of photons exiting the tissue

$$(\begin{array}{c} x_{s}\\ \theta_{x,s} \end{array})=S(\begin{array}{c} x\\ \theta_{x} \end{array}),\;(\begin{array}{c} y_{s}\\ \theta_{y,s} \end{array})=S(\begin{array}{c} y\\ \theta_{y} \end{array}).$$ (2)

The diffuse reflectance is calculated for each pixel, either on the tissue surface or on the camera sensor^45^^,^^46^

$$\phi (x,y,\lambda )=\frac{\overset{N_{\text{photons}}}{\underset{j=1}{\sum }}w_{j}(x,y,\lambda )}{N_{\text{photons}}.A_{p}},$$ (3)

where $N_{\text{photons}}$ is the number of packets of photons detected at the pixel (x,y), $A_{p}$ is the area (in ${\mathrm{mm}}^{2}$) of the pixel, and $w_{j}$ is the weight of the detected packet of photons j defined by

$$w_{j}(x,y,\lambda )=\Pi_{i=1}^{N_{\text{regions}}}\;\exp(-\mu_{a,i}(\lambda ).{\mathrm{ppl}}_{i,j}(x,y,\lambda )).$$ (4)

$N_{\text{regions}}$ is the number of regions modeled in the tissue (grey matter and large and small blood vessels), $\mu_{a,i}(\lambda )$ (in ${\mathrm{mm}}^{-1}$) is the absorption coefficient for the wavelength λ of the region i, and ${\mathrm{ppl}}_{i,j}(x,y,\lambda )$ is the partial path length (in mm) of the detected packet of photon j that traveled in region i for wavelength λ. The mean path length of traveled photons in the tissue is calculated in Eq. (5)^46^

$$L(x,y)=\frac{\overset{N_{\text{photons}}}{\underset{j=1}{\sum }}\overset{N_{\text{regions}}}{\underset{i=1}{\sum }}{\mathrm{ppl}}_{i,j}(x,y,\lambda ).w_{j}(x,y,\lambda )}{\overset{N_{\text{photons}}}{\underset{j=1}{\sum }}w_{j}(x,y,\lambda )}.$$ (5)

Finally, we applied an adaptive non-local means filter on reconstructed diffuse reflectance and mean path length images for each wavelength. This operation aims to improve the signal-to-noise ratio of the Monte Carlo simulations.^47^ Finally, we performed a linear interpolation for each pixel of the diffuse reflectance and mean path length images on the wavelength range 400 to 1000 nm by steps of 1 nm.

### 2.2 Identification of the Optimal Wavelength for Hemodynamic and Metabolic Monitoring

Using the digital instrument simulator (see Sec. 2.1), we developed an optimization procedure based on a genetic algorithm (differential evolution) to identify the best wavelength combination in the visible and near-infrared range to minimize the quantification error (root mean square error in M) in ${\mathrm{HbO}}_{2}$, Hb, oxCCO, oxCytb, and oxCytc compared with ground truth values, see Fig. 6.

#### 2.2.1 Model of cerebral activation

**Fig. 5:**
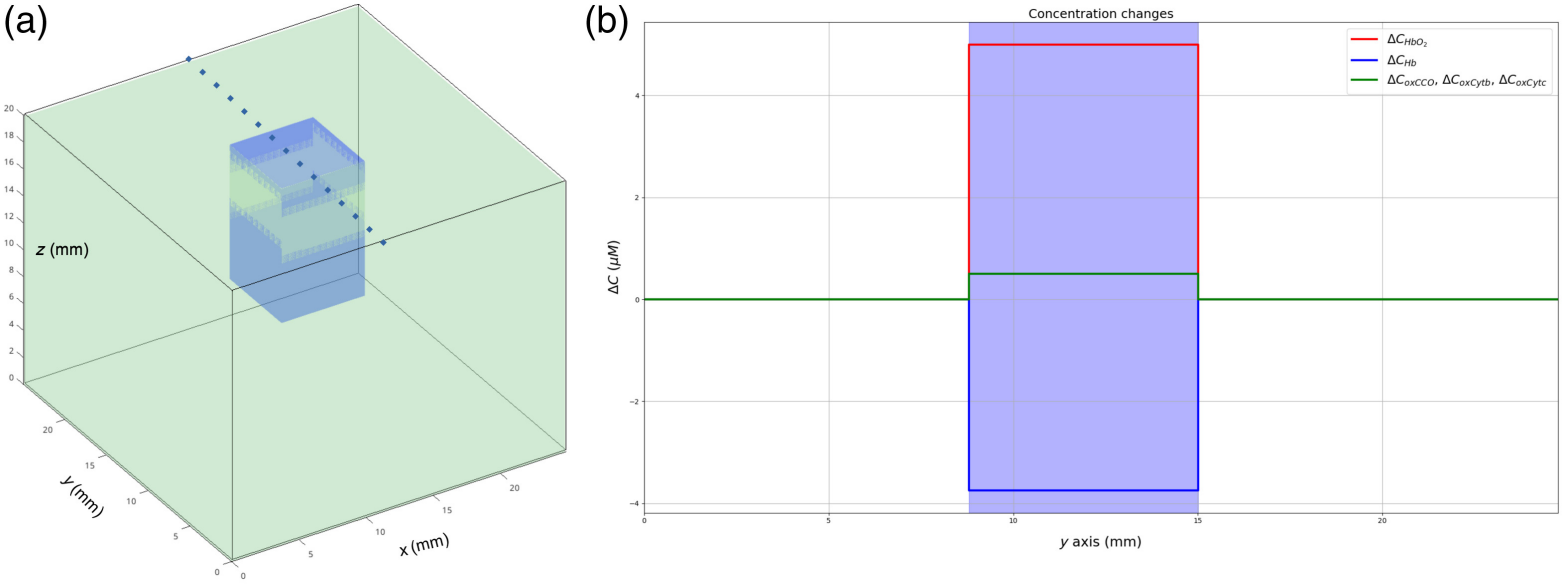
Monte Carlo model used for the optimization procedure. (a) Volume of non-activated grey matter (green) and activated grey matter (blue). (b) Spatial profile of the modeled concentration changes $\Delta C_{\mathrm{GT}}$ indicated by the dotted line in panel (a).

Data used for the optimization procedure were generated with the digital instrument simulator. A simple model was considered, which includes non-activated and activated grey matter, see Fig. 5(a). In Fig. 5(b), we modeled a cerebral activity in the activated grey matter by changing the concentration of chromophores relative to their nominal values. The modeled concentration changes that are the ground truth in the optimization procedure are indicated $\Delta C_{\mathrm{GT}}$ in the rest of the paper. A simple temporal perturbation of $\mu_{a}$ was performed: rest (nominal values, see Table 2) and activity (nominal value +ΔC). The ΔC values were +5 and −3.75  μM for ${\mathrm{HbO}}_{2}$ and Hb,^9^^,^^48^ respectively, and +0.5  μM for oxCCO,^49^ oxCytb, and oxCytc, mirrored by concentration changes of −0.5  μM for redCCO, redCytb, and redCytc (the concentration of cytochromes does not change during the cerebral activity).

#### 2.2.2 Optimization procedure

**Fig. 6:**
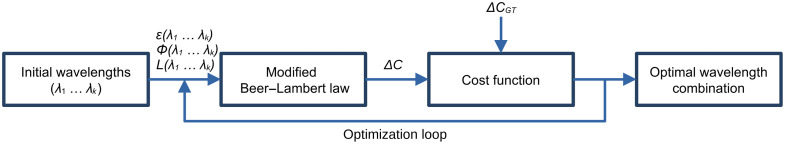
Flowchart of the optimization method, including definitions of the variables used in the procedure.

The most straightforward method to determine k optimal wavelengths for cerebral monitoring is to test all possible k-element combinations out of 601 wavelengths (from 400 to 1000 nm) and to identify the combination that produces the smallest estimation errors when compared with the ground truth $\Delta C_{\mathrm{GT}}$. As the investigated spectral range is large, such an exhaustive search results in a large time complexity. Thus, we proposed to use a genetic algorithm^50^ to determine the k optimal wavelengths through an optimization procedure, see Fig. 6. We used the differential evolution method that optimizes a problem by iteratively trying to improve a candidate solution with regard to the ground truth. We used the function differential evolution from the Python library Scipy (v1.10.0).^51^

The optimization procedure consists of finding the best k-element combinations out of 601 wavelengths that minimize a cost function. This function takes as input a wavelength array, with values that are limited to the simulated spectral range (between 400 and 1000 nm). The cost function returns the root mean square error RMSE (in M) computed between noisy concentration changes measured with the modified Beer–Lambert law and the ground truth $\Delta C_{\mathrm{GT}}$

$$\mathrm{RMSE}=\sqrt{\overset{N_{\text{Noise}}}{\underset{i=1}{\sum }}\frac{(\Delta C_{\mathrm{mes}}^{\text{Noise}}-\Delta C_{\mathrm{GT}}{)}^{2}}{N_{\text{Noise}}}},$$ (6)

where $N_{\text{Noise}}=1000$ is the number of iterations for the noise addition. Noise was added to the simulated intensities to identify the k-wavelengths the most robust to the noise. The intensities were converted into concentration changes $\Delta C_{\mathrm{mes}}^{\text{Noise}}$ measured with the modified Beer–Lambert law

$$(\begin{array}{c} \Delta A(\lambda_{1}{)}^{\text{Noise}}\\ \vdots \\ \Delta A(\lambda_{1}{)}^{\text{Noise}} \end{array})=(\begin{array}{ccccc} L(\lambda_{1}).\epsilon_{{\mathrm{HbO}}_{2}}(\lambda_{1}) & L(\lambda_{1}).\epsilon_{\mathrm{Hb}}(\lambda_{1}) & L(\lambda_{1}).\epsilon_{\mathrm{ox}\text{-}\mathrm{redCCO}}(\lambda_{1}) & L(\lambda_{1}).\epsilon_{\mathrm{ox}\text{-}\mathrm{redCytb}}(\lambda_{1}) & L(\lambda_{1}).\epsilon_{\mathrm{ox}\text{-}\mathrm{redCytc}}(\lambda_{1})\\ \vdots & \vdots & \vdots & \vdots & \vdots \\ L(\lambda_{k}).\epsilon_{{\mathrm{HbO}}_{2}}(\lambda_{k}) & L(\lambda_{k}).\epsilon_{\mathrm{Hb}}(\lambda_{k}) & L(\lambda_{k}).\epsilon_{\mathrm{ox}\text{-}\mathrm{redCCO}}(\lambda_{k}) & L(\lambda_{k}).\epsilon_{\mathrm{ox}\text{-}\mathrm{redCytb}}(\lambda_{k}) & L(\lambda_{k}).\epsilon_{\mathrm{ox}\text{-}\mathrm{redCytc}}(\lambda_{k}) \end{array})\times (\begin{array}{c} \Delta C_{{\mathrm{HbO}}_{2}}^{\text{Noise}}\\ \Delta C_{\mathrm{Hb}}^{\text{Noise}}\\ \Delta C_{\mathrm{oxCCO}}^{\text{Noise}}\\ \Delta C_{\mathrm{oxCytb}}^{\text{Noise}}\\ \Delta C_{\mathrm{oxCytc}}^{\text{Noise}} \end{array}).$$ (7)

In Eq. (7), $\epsilon_{n}(\lambda_{1})$ is the molar extinction coefficient of the chromophore n (in $M^{-1}.{\mathrm{mm}}^{-1}$), see Fig. S1 in the Supplementary Material. $\Delta A(\lambda_{1}{)}^{\text{Noise}}$ is the noisy attenuation change measured for $\lambda_{1}$, such as

$$\Delta A(\lambda_{1}{)}^{\text{Noise}}=\log_{10}(\frac{\phi_{\text{rest}}(\lambda_{1})+\gamma }{\phi_{\text{activity}}(\lambda_{1})+\gamma }),$$ (8)

where γ is a zero-mean Gaussian noise with a standard deviation that was the same for each wavelength: $\sigma =\frac{\phi_{\text{rest}}}{\mathrm{SNR}}$. With $\phi_{\text{rest}}$, the diffuse reflectance simulated during rest and SNR=400 the signal-to-noise ratio of the instrument. This value corresponds to the experimental devices we used in a previous study,^22^ but this could be adapted for another experimental device. The concentration changes were obtained through matrix inversion in the least square sense.

## 3 Results

### 3.1 Digital Instrument Simulator

**Fig. 7:**
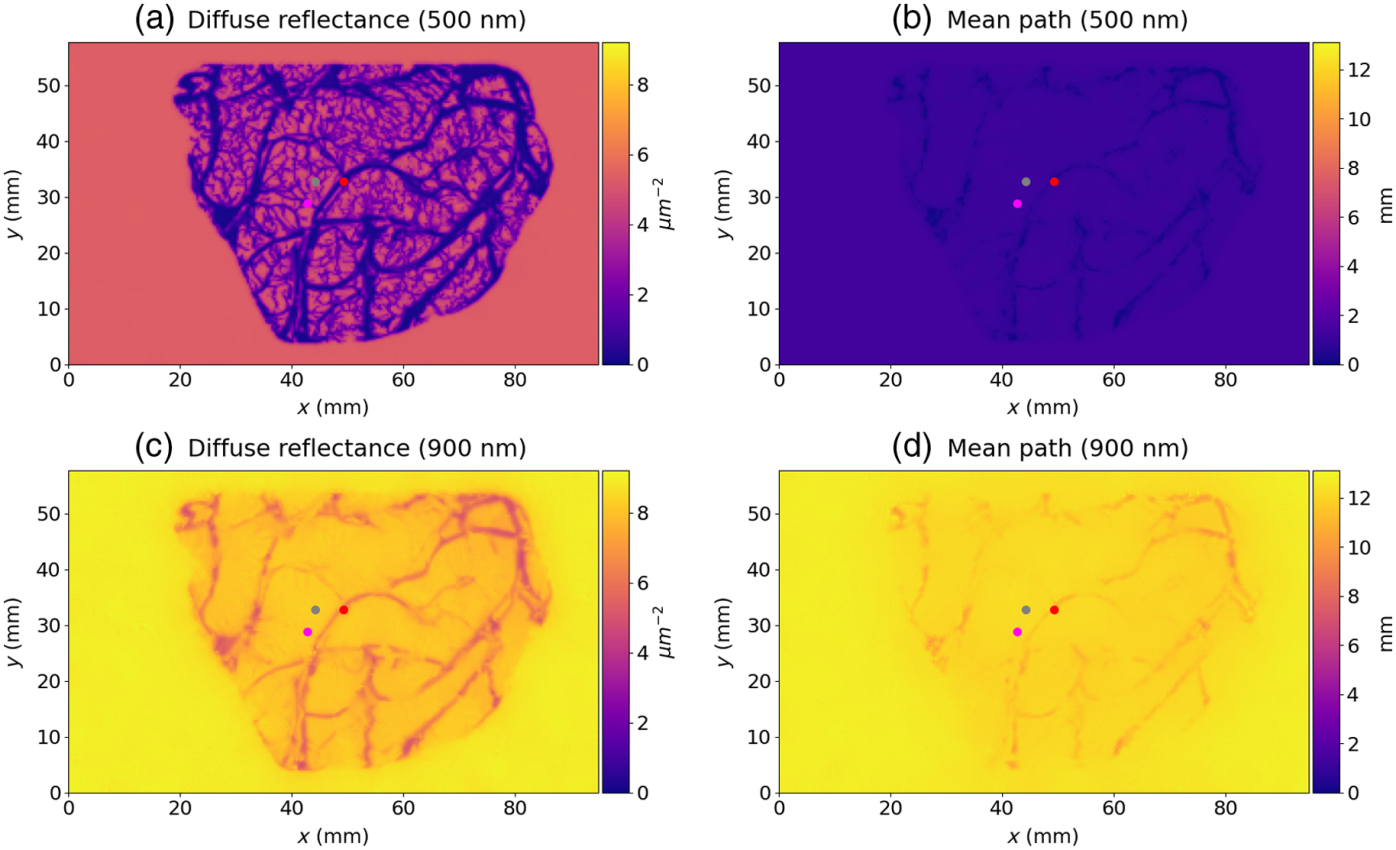
Images of diffuse reflectance and mean path length reconstructed with a 5×5 binning at 500 and 900 nm. (a) Diffuse reflectance image simulated at 500 nm. (b) Mean path image simulated at 500 nm. (c) Diffuse reflectance image simulated at 900 nm. (d) Mean path image simulated at 900 nm. The large and small blood vessels are considered arteries. The red, grey, and magenta points indicate the position of points for a large blood vessel, for grey matter, and for a small blood vessel, respectively.

The digital instrument simulator aimed to simulate hyperspectral images of the exposed brain cortex for 601 wavelengths (between 400 and 1000 nm). In Fig. 7, we represented the images of diffuse reflectance and mean path length at 500 and 900 nm reconstructed at the surface of the tissue considering that large and small blood vessels are arteries. The images were reconstructed with a 5×5 binning, which leads to a resolution of 365  μm. The red, grey, and magenta points indicate the position of points for a large blood vessel, for grey matter, and for a small blood vessel, respectively.

**Fig. 8:**
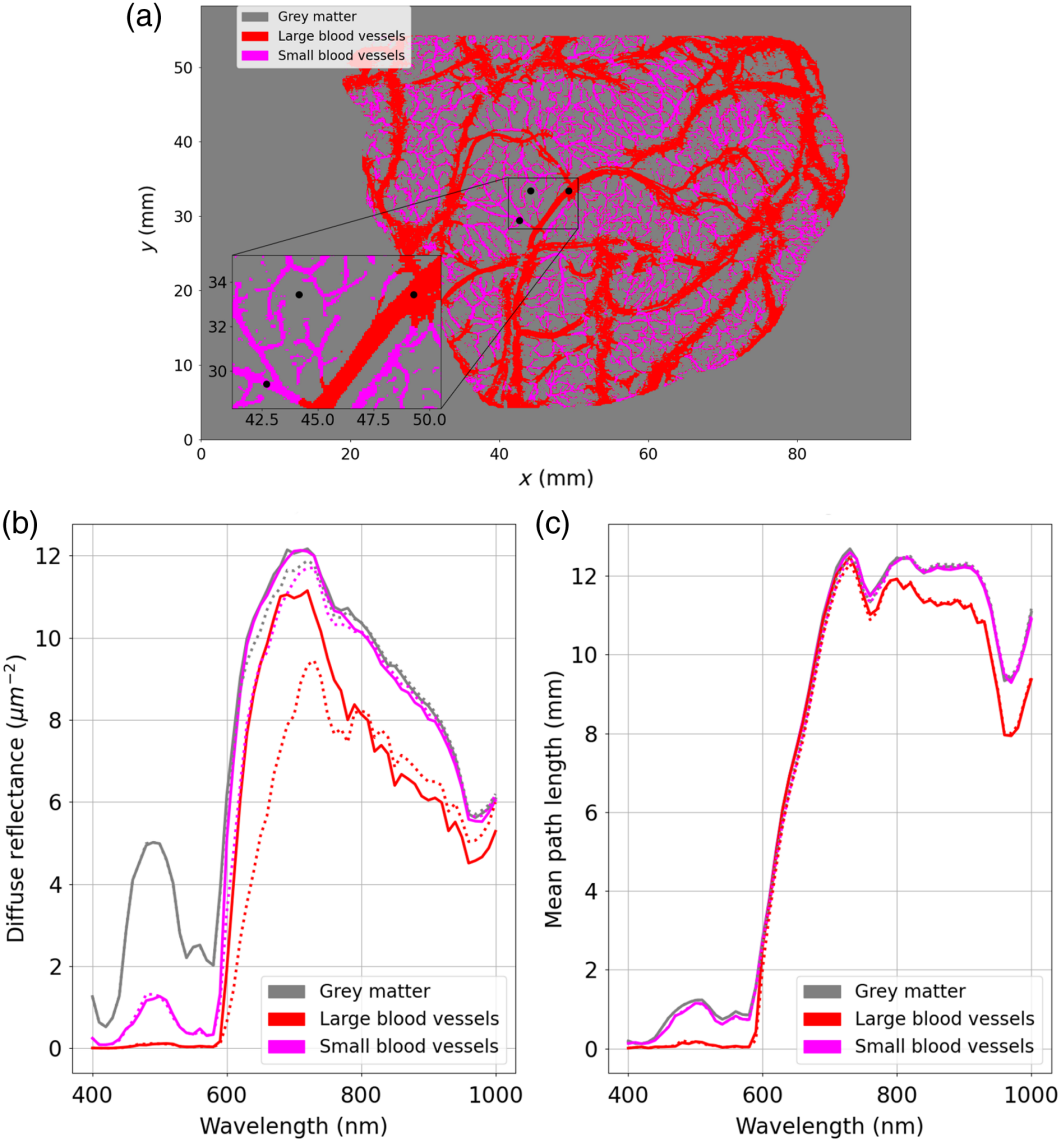
Diffuse reflectance and mean path length spectra measured on grey matter, a large blood vessel, and a small blood vessel. (a) Image of the cerebral cortex segmented into three classes (grey matter and large and small blood vessels). The three black points indicate the position of points for a large blood vessel, for grey matter, and for a small blood vessel. (b) Diffuse reflectance spectra collected at the location of the three black points. (c) Mean path length spectra collected at the location of the three black points. Solid and dotted lines indicate that large and small blood vessels are considered arteries and veins, respectively.

In Fig. 8, we represented the diffuse reflectance and mean path length spectra measured for the three points identified in Fig. 7. Solid and dotted lines indicate that large and small blood vessels are considered arteries and veins, respectively. For both cases, we can observe the effect of the blood vessels on the simulated quantities. For wavelengths lower than 600 nm, the diffuse reflectance was almost $0\;\mu m^{-2}$ on the large blood vessels; this means that hardly any photons left the large blood vessel. Diffuse reflectance values increased when measuring small blood vessels, indicating a greater contribution from outgoing photons. The higher values can be found for grey matter regions. For wavelengths higher than 600 nm, diffuse reflectance values were almost the same for grey matter and small blood vessels, and lower values can be found at the level of the large blood vessels. The mean path length spectra measured for grey matter and the small blood vessels were almost the same for all wavelengths, but lower values were obtained for the large blood vessels. We can observe that the values of the mean path length are not impacted if all vessels are considered either veins or arteries. However, we can observe differences among the diffuse reflectance spectra measured at the level of the large blood vessels for wavelengths higher than 600 nm. There is a drop in intensity in the red and near infrared for veins compared with arteries. This corresponds to the fact that veins appear darker or bluer than arteries.

### 3.2 Identification of the Optimal Wavelength for Hemodynamic and Metabolic Monitoring

**Fig. 9:**
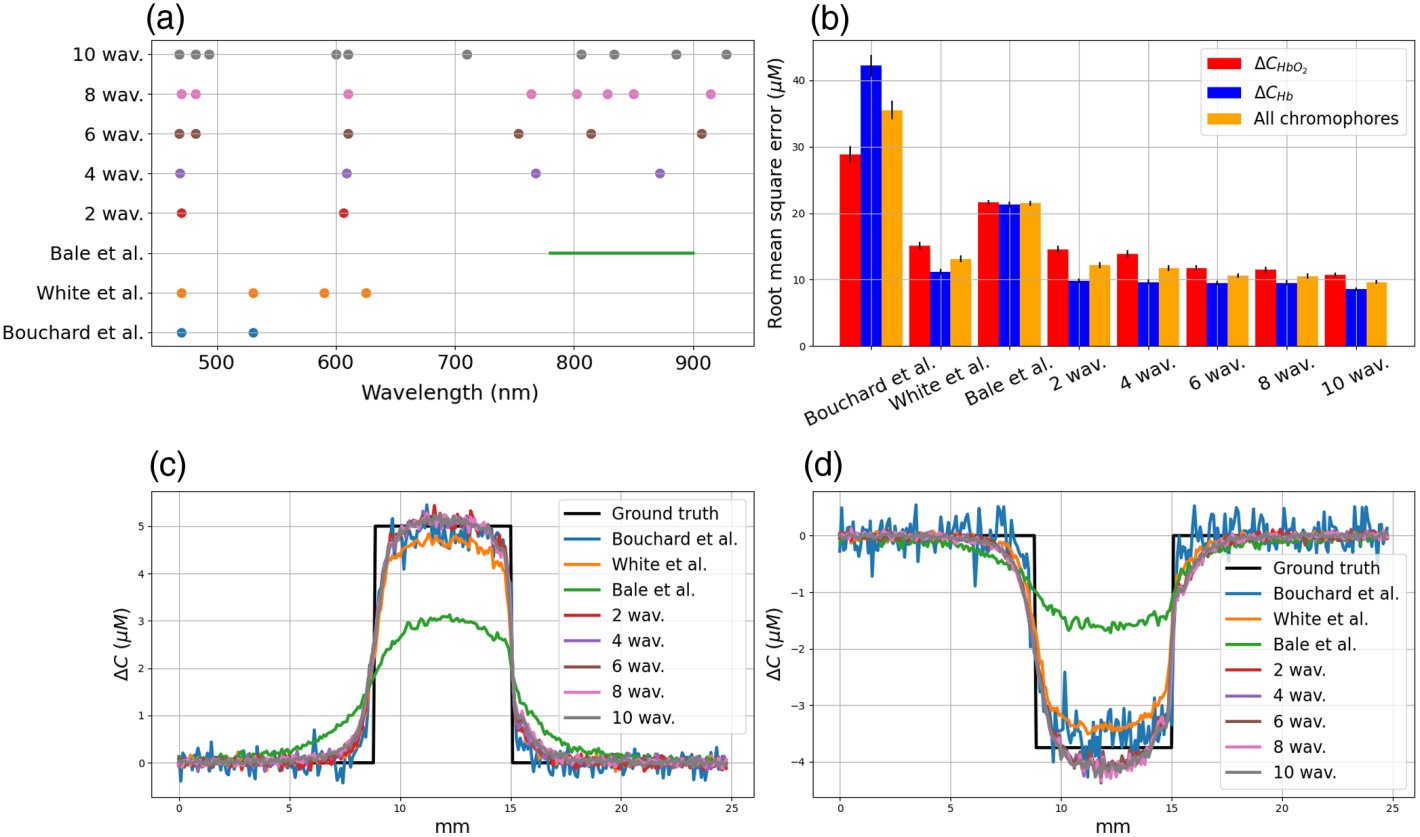
(a) Optimal wavelength for monitoring $C_{{\mathrm{HbO}}_{2}}$ and $C_{\mathrm{Hb}}$ changes in activated grey matter. (b) Quantification errors (in μM). (c) and (d) Concentration changes in ${\mathrm{HbO}}_{2}$ and Hb, respectively. Several configurations are represented: two wavelengths proposed by Bouchard et al.,^29^ four wavelengths proposed by White et al.,^30^ a broadband spectra proposed by Bale et al.,^14^^,^^17^ and the combination of 2, 4, 6, 8, and 10 wavelengths identified with our optimization procedure.

In Fig. 9, we represented the optimal combination of 2, 4, 6, 8, and 10 wavelengths for monitoring $C_{{\mathrm{HbO}}_{2}}$ and $C_{\mathrm{Hb}}$ changes in activated grey matter. These wavelengths are represented in Fig. 9(a) by dots. We also represented the configuration of two wavelengths used by Bouchard et al.,^29^ the four wavelengths used by White et al.^30^ and the broadband spectral range between 780 and 900 nm used by Bale et al.^14^^,^^17^ and Arifler et al.^31^ The quantification errors related to these configurations are plotted in Fig. 9(b). The bars represent the RMSE averaged over 1000 noisy measurements, see Eq. (6), and the vertical line represents the standard deviation in the error. Finally, the concentration changes in $\Delta C_{{\mathrm{HbO}}_{2}}$ and $\Delta C_{\mathrm{Hb}}$ averaged over the 1000 noisy measurements are plotted in Figs. 9(c) and 9(d), respectively.

Contrary to the configurations proposed by Bouchard et al. and White et al., the wavelengths identified with the optimization procedure do not include the hemoglobin isobestic point at 530 nm. The wavelengths were located in the visible and near-infrared range. Wavelengths were identified before 500 nm, where ${\mathrm{HbO}}_{2}$ absorption predominates, between 590 and 730 nm where Hb absorption predominates and on either side of the isobestic point at 800 nm. The minimum errors in $\Delta C_{{\mathrm{HbO}}_{2}}$ and $\Delta C_{\mathrm{Hb}}$ are obtained with the combination of 10 wavelengths calculated with our optimization procedure. Among the literary configurations, White’s spectral configuration minimizes errors in $\Delta C_{{\mathrm{HbO}}_{2}}$ and $\Delta C_{\mathrm{Hb}}$. Errors in $\Delta C_{{\mathrm{HbO}}_{2}}$ and $\Delta C_{\mathrm{Hb}}$ obtained with the optimal group of 10 wavelengths were 29% and 23% lower than those obtained with White’s spectral configuration. The mean and standard deviation of the RMSE decrease slightly as more spectral bands are considered. For all wavelengths, the standard deviation and the mean RMSE are lower than those obtained with White’s spectral configuration.

**Fig. 10:**
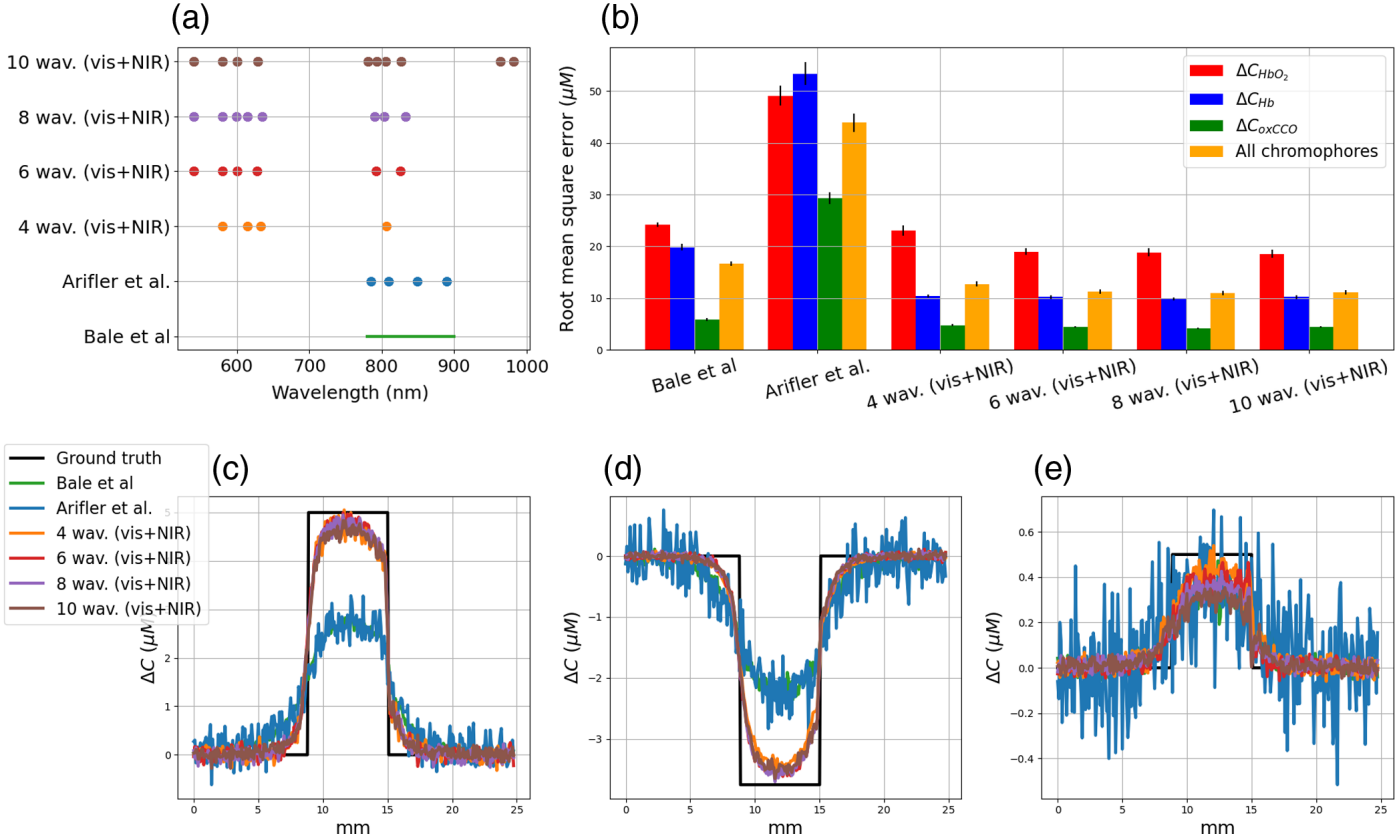
(a) Optimal wavelength for monitoring $C_{{\mathrm{HbO}}_{2}}$, $C_{\mathrm{Hb}}$, and $C_{\mathrm{oxCCO}}$ changes in activated grey matter. (b) Quantification errors (in μM). (c), (d), and (e) Concentration changes in ${\mathrm{HbO}}_{2}$, Hb, and oxCCO, respectively. Several configurations are represented: four wavelengths proposed by Arifler et al.,^31^ a broadband spectra proposed by Bale et al.,^14^^,^^17^ and the combination of 4, 6, 8, and 10 wavelengths identified with our optimization procedure.

In Fig. 10, we represented the optimal combination of 4, 6, 8, and 10 wavelengths for monitoring $C_{{\mathrm{HbO}}_{2}}$, $C_{\mathrm{Hb}}$, and $C_{\mathrm{oxCCO}}$ changes in activated grey matter. These wavelengths are represented in Fig. 10(a) by dots. We also represented the configuration of four wavelengths proposed by Arifler et al.^31^ and the broadband spectral range used by Bale et al.^14^^,^^17^ The quantification errors are represented in Fig. 10(b) and the measurements of $\Delta C_{{\mathrm{HbO}}_{2}}$, $\Delta C_{\mathrm{Hb}}$, and $\Delta C_{\mathrm{oxCCO}}$ are plotted in Figs. 10(c), 10(d), and 10(e), respectively.

The minimum errors in $\Delta C_{{\mathrm{HbO}}_{2}}$, $\Delta C_{\mathrm{Hb}}$, and $\Delta C_{\mathrm{oxCCO}}$ are obtained with the combination of 10 wavelengths calculated with our optimization procedure. With this digital instrument simulator, we modeled an exposed cortex. The simulated light is not absorbed by the skin or the skull of the patient, and the visible light can be collected by the camera. Thus, the optimization procedure can take profit of the peaks of oxidized and reduced CCO extinction spectra in the visible and the near-infrared range to monitor the three chromophores. The errors in $\Delta C_{{\mathrm{HbO}}_{2}}$, $\Delta C_{\mathrm{Hb}}$, and $\Delta C_{\mathrm{oxCCO}}$ obtained with this configuration were 23%, 48%, and 23% lower than those obtained with broadband spectra between 780 and 900 nm, respectively. For all chromophores, the standard deviation of the RMSE decreases as more spectral bands are considered. For $\Delta C_{{\mathrm{HbO}}_{2}}$, the mean RMSE decreases slightly with the number of wavelengths, whereas the mean errors for $\Delta C_{\mathrm{Hb}}$ and $\Delta C_{\mathrm{oxCCO}}$ remain constant. For all wavelengths, the mean RMSE is lower than that computed with Bale’s spectral configuration. For $\Delta C_{\mathrm{Hb}}$ and $\Delta C_{\mathrm{oxCCO}}$, the standard deviation is less than or equal to that obtained with Bale’s configuration. However, for $\Delta C_{{\mathrm{HbO}}_{2}}$, the standard deviation is higher than that obtained with Bale’s configuration.

**Fig. 11:**
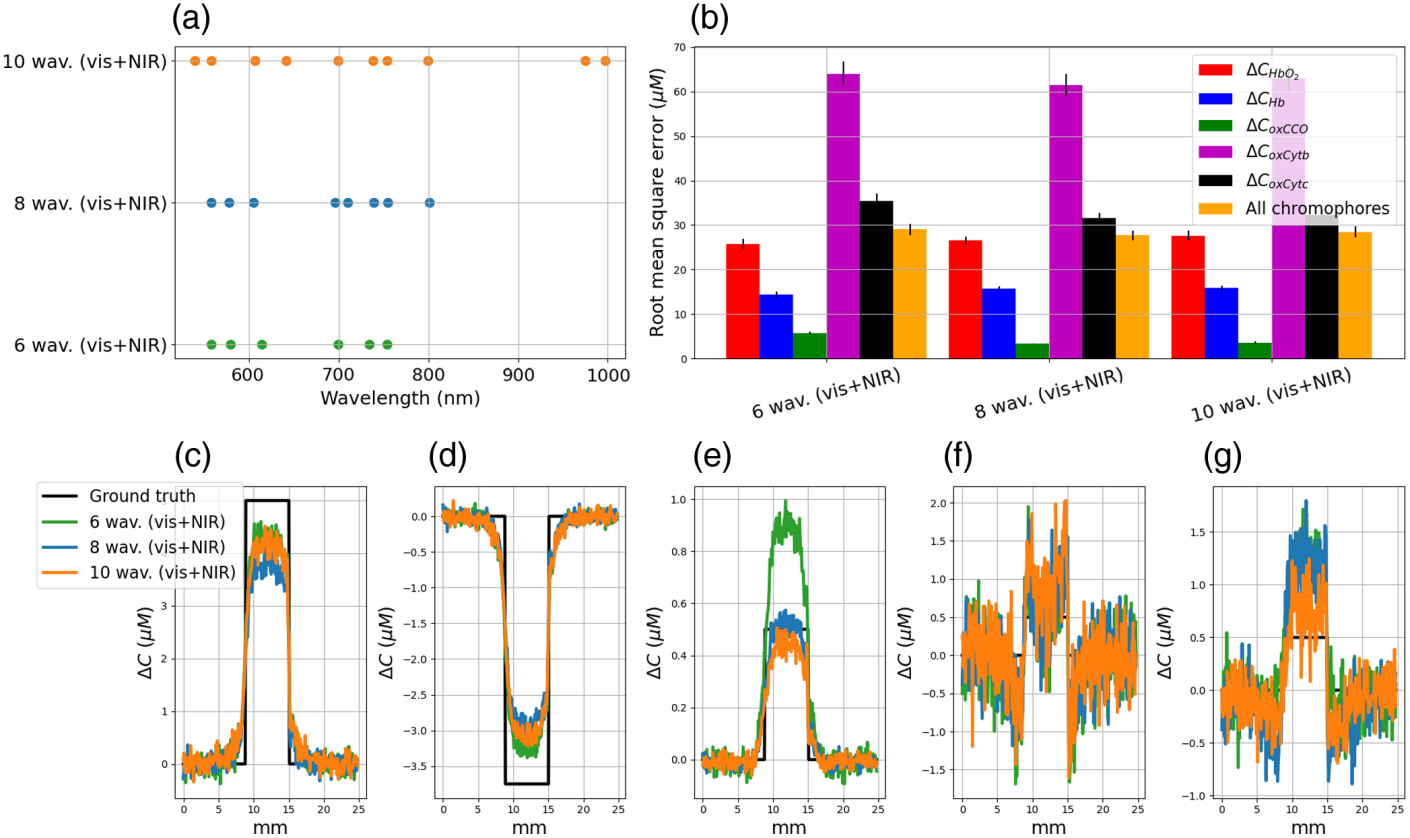
(a) Optimal wavelength for monitoring $C_{{\mathrm{HbO}}_{2}}$, $C_{\mathrm{Hb}}$, $C_{\mathrm{oxCCO}}$, $C_{\mathrm{oxCytb}}$, and $C_{\mathrm{oxCytc}}$ changes in activated grey matter. (b) Quantification errors (in μM). (c), (d), (e), (f), and (g) Concentration changes in ${\mathrm{HbO}}_{2}$, Hb, oxCCO, oxCytb, and oxCytc, respectively.

In Fig. 11, we represented the optimal combination of 6, 8, and 10 wavelengths for monitoring $C_{{\mathrm{HbO}}_{2}}$, $C_{\mathrm{Hb}}$, $C_{\mathrm{oxCCO}}$, $C_{\mathrm{oxCytb}}$, and $C_{\mathrm{oxCytc}}$ changes in activated grey matter. These wavelengths are represented in Fig. 11(a) by dots. The quantification errors are represented in Fig. 11(b), and the concentration changes are plotted in Figs. 11(c), 11(d), 11(e), 11(f), and 11(g).

The minimum errors to resolve all chromophores are obtained with the combination of 8 and 10 wavelengths. It is interesting to note that the use of a larger number of wavelengths (10) does not significantly reduce quantification errors. With the use of 6, 8, and 10 optimal wavelengths, the total quantification errors were 29, 28, and 28  μM, respectively. The errors in $\Delta C_{{\mathrm{HbO}}_{2}}$ and $\Delta C_{\mathrm{Hb}}$ were higher than those obtained in Fig. 10. However, with the consideration of cytochromes b and c in the modified Beer–Lambert law, the errors in $\Delta C_{\mathrm{oxCCO}}$ were 19% lower than those obtained in Fig. 10. The noise in the measurements of $\Delta C_{\mathrm{oxCytb}}$ and $\Delta C_{\mathrm{oxCytc}}$ is very important, making it difficult to differentiate between activated and non-activated grey matter.

**Fig. 12:**
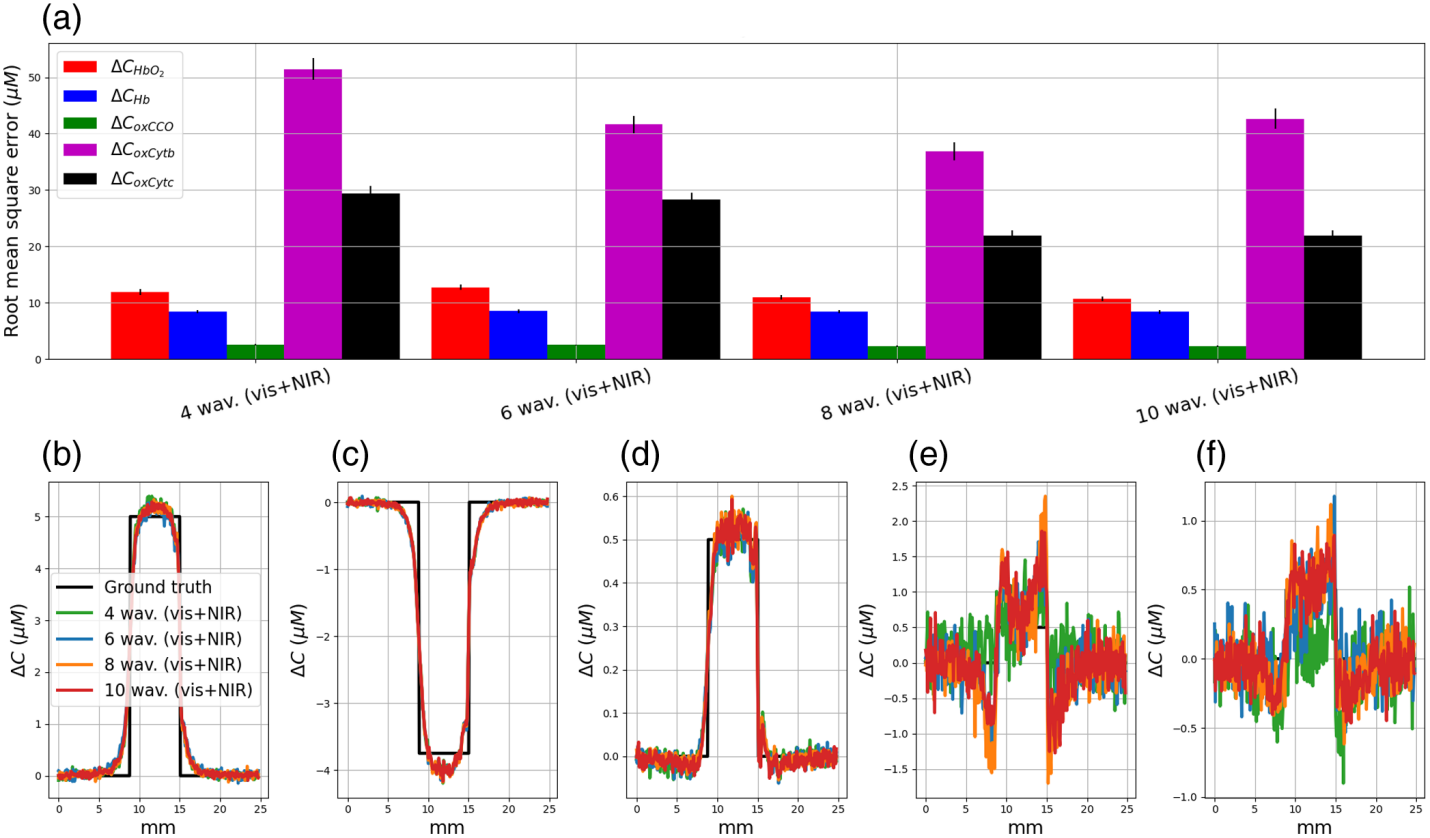
Quantification errors (a) and concentration changes obtained with the optimal combination of 4, 6, 8, and 10 wavelengths for a separate quantification. The concentration changes in $C_{{\mathrm{HbO}}_{2}}$, $C_{\mathrm{Hb}}$, $C_{\mathrm{oxCCO}}$, $C_{\mathrm{oxCytb}}$, and $C_{\mathrm{oxCytc}}$ are plotted in panels (b), (c), (d), (e), and (f), respectively.

As proposed in our previous study,^22^ we can also identify different combinations of wavelengths to independently quantify the concentration changes in ${\mathrm{HbO}}_{2}$, Hb, oxCCO, oxCytb, and oxCytc, see Fig. 12. For instance, if we want to identify the optimal wavelength to resolve $C_{\mathrm{oxCytb}}$ changes only, the optimization procedure can be run to minimize $\Delta C_{\mathrm{oxCytb}}$ values by only considering three chromophores in the modified Beer–Lambert law [${\mathrm{HbO}}_{2}$, Hb, and oxCytb, see Eq. (7)].

With this approach, we represented in Fig. 12(a) the quantification errors obtained with the optimal wavelength combination to separately resolve ${\mathrm{HbO}}_{2}$, Hb, oxCCO, oxCytb, and oxCytc changes. We also plotted the concentration changes in ${\mathrm{HbO}}_{2}$, Hb, oxCCO, oxCytb, and oxCytc in Figs. 12(b), 12(c), 12(d), 12(e), and 12(f). In Fig. S2 in the Supplementary Material, we represented the optimal combinations of 4, 6, 8, and 10 wavelengths to separately resolve ${\mathrm{HbO}}_{2}$, Hb, oxCCO, oxCytb, and oxCytc changes. The separate optimization procedure leads to different spectral configurations for each chromophore.

For all chromophores (expect oxCytb), the mean RMSE decreases with the number of wavelengths. We obtained smaller quantification errors when we applied the optimization procedure on each chromophore separately than when we tried to resolve all the chromophores at the same time, see Table 3.

**Table 3:** Average of the quantification errors (RMSE in μMol) obtained with two Beer–Lambert systems proposed in the literature and optimal combination of six wavelengths.

Modified Beer–Lambert law system	${\mathrm{HbO}}_{2}$	Hb	oxCCO	oxCytb	oxCytc
Broadband spectra (780 to 900 nm)^14^^,^^17^	24.1	19.8	5.8	—	—
White’s configuration^30^	15.2	11.2		—	—
${\mathrm{HbO}}_{2}$, Hb, and oxCCO (6 wavelengths), see Fig. 10	19.1	10.4	4.4	—	—
All chromophores at the same time (6 wavelengths), see Fig. 11	25.9	14.4	3.6	64.6	35
All chromophores separately (6×5 wavelengths), see Fig. 12	12.7	8.5	2.5	42.1	28.3

## 4 Discussion

We presented a digital instrument simulator to optimize the development of hyperspectral systems for intraoperative brain mapping studies. The code is publicly available on GitHub. This simulator is based on a realistic digital phantom of an exposed cortex computed with white Monte Carlo simulations. We developed C++ software to reconstruct temporal perturbations of the absorption coefficient using only one simulation. Using this Monte Carlo framework, we also proposed an optimization procedure based on the genetic algorithm to identify the best wavelength combinations in the visible and near-infrared range to quantify changes in ${\mathrm{HbO}}_{2}$, Hb, oxCCO, oxCytb, and oxCytc.

This digital instrument simulator has been developed for intraoperative brain mapping studies. However, the code could be easily adapted for other studies. For an organ other than the brain, the segmentation step will certainly have to be adapted (segmentation method, number of classes), as well as the optical properties (see Table 1) and the chemical composition (see Table 2) for each class of the modeled tissue.

### 4.1 Digital Instrument Simulator

The digital instrument allows the modeling of intensity maps collected by a camera sensor as well as the estimation of the mean path length of traveled photons through the tissue. This framework could be used to improve clinical and preclinical optical devices for brain mapping applications. In these studies, the modified Beer–Lambert law is used to monitor chromophore changes in an animal^29^^,^^30^ or a human^7^^,^^9^^,^^11^^,^^52^ brain. However, measurements are highly subject to quantification errors if incorrect path lengths are used to resolve chromophore changes. This could be the case in a lot of applications where the inhomogeneities of the optical properties are not taken into consideration. Indeed, a single path length is considered for the whole cerebral cortex, which is usually estimated with a homogeneous volume of grey matter with Monte Carlo simulations^29^ or using the analytical solution to the diffusion approximation of the radiative transfer equation in a semi-infinite geometry.^30^^,^^52^ In our study, we proposed to take into account the inhomogeneities of the optical properties with a pixel-wise estimation of the mean path length. As we can see on Figs. 7 and 8, the blood vessels have a great impact on the path length. For large blood vessels having a 2-mm diameter, the path length is almost null in the visible spectra (below 600 nm) and is lower than the path length estimated for grey matter in the red and near-infrared range. However, the path length measured at the level of the small blood vessels (≈0.2  mm) is almost the same as that measured at the level of the grey matter. This means that the detected packets of photons are mostly propagated in the grey matter under these small blood vessels. This result is consistent with the results presented by Giannoni et al.^24^ and indicates that large blood vessels have a large impact on the path length. We also observed that there were no significant differences between the mean path length measured on arteries and veins. Indeed, when comparing the absorption coefficients of the artery and the vein ($\overset{¯}{\mu_{a,\text{Artery}}-\mu_{a,\text{Vein}}}=-15\;{\mathrm{cm}}^{-1}$), we do not observe as great a difference as that between the artery and the grey matter ($\overset{¯}{\mu_{a,\text{Artery}}-\mu_{a,\text{Grey matter}}}=172\;{\mathrm{cm}}^{-1}$) or the vein and the grey matter ($\overset{¯}{\mu_{a,\text{Artery}}-\mu_{a,\text{Grey matter}}}=188\;{\mathrm{cm}}^{-1}$, with $\overset{¯}{\mu_{a}}$ the average absorption coefficient over the wavelength range 400 to 1000 nm).

The segmentation step in the digital instrument simulator could also be improved, see Fig. 1 and Sec. 2.1.2. Segmentation is based on morphological operations calculated in a mask delimiting the surgical window. The latter was created manually. This manual operation has been chosen because automatic thresholding, using Otsu’s method^53^ for example, does not allow the cerebral cortex to be identified repeatedly. In some patients, bleeding around the edges of the surgical window or bloody compresses prevents the cerebral cortex from being correctly identified. To overcome this limitation, segmentation methods based on deep learning (U-Net)^54^ could be used for segmenting the blood vessels and delineate the contour of the surgical window. For the moment, this approach has not been implemented because we do not have enough data to train the neural network. In this study, we modeled a perfect optical device. The real spectral characteristics of the light source and the sensor could be integrated into the digital simulator by normalizing the estimated quantities with spectral sensitivity curves of the light sources and the camera, as proposed in our previous study.^22^ We modeled a real sensor in terms of resolution and definition, but this could be improved with the conversion of the diffuse reflectance into sensor intensities by considering parameters of the optics systems^55^ (lens magnification, lens transmission, quantum efficiency). We can also integrate the effect of the lenses on the image creation such as geometric distortion, vignetting, or chromatic aberrations. A lens design model can be used for this purpose.^56^ Using this digital instrument simulator, we modeled a simple cerebral activation by changing the concentrations of the chromophores in a portion of activated grey matter (see Fig. 5). However, we do not model the complex physiologic events related to the neuro-vascular coupling. To answer this limitation, we plan to model the spatio-temporal concentration changes in arteriole, capillary, and venous compartments related to blood volume, flow velocity, and oxygen consumption with the implementation of the dynamic model proposed by Fantini.^23^

Although the strength of this digital instrument is to use the white Monte Carlo approach, this method has also a limitation, which is the size of the generated files. Indeed, for one wavelength, 5 GB of data is generated, which leads to 305 GB for the 61 wavelengths modeled in our study. To reduce the memory size, the absorption coefficients need to be fixed in the modeled tissue to directly estimate the radiative quantities. However, with this approach, we cannot reconstruct the temporal changes of the absorption coefficient, so a simulation has to be performed per wavelength and per temporal index, which increases drastically the computation time.

### 4.2 Identification of the Optimal Wavelength for Hemodynamic and Metabolic Monitoring

Using the data obtained with the digital instrument simulator, we proposed an optimization procedure based on the genetic algorithm to identify the best wavelength combinations in the visible and near-infrared range to quantify changes in ${\mathrm{HbO}}_{2}$, Hb, oxCCO, oxCytb, and oxCytc. We added noise to the simulated quantities to identify the wavelength that is the most robust to noise.

For hemodynamic monitoring, see Fig. 9, we can see that the optimal groups of 2, 4, 6, 8, or 10 wavelengths aim to reduce the quantification errors in $\Delta C_{{\mathrm{HbO}}_{2}}$ and $\Delta C_{\mathrm{Hb}}$ compared with that obtained with the configurations proposed in the literature. Contrary to the spectral configurations proposed by Bouchard et al.^29^ and White et al.,^30^ the wavelengths identified with the optimization procedure do not include the hemoglobin isobestic point at 530 nm. The wavelengths were located in the visible and near-infrared range, where ${\mathrm{HbO}}_{2}$ or Hb absorption predominates. This could be explained by the addition of noise in the optimization procedure, which makes it difficult to interpret the attenuation changes measured at the isobestic points. Moreover, the optimization procedure identifies the best wavelengths but does not take into account the bandwidth of the light source spectra in hyperspectral devices based on spectral-scanning technology.^28^ Taking into account a spectral bandwidth could modify the value of the central wavelengths by a few nanometers but would not introduce changes greater than the spectral bandwidth.

The optimization procedure took profit of the peaks of oxidized and reduced cytochrome extinction spectra in the visible and the near-infrared range to monitor hemodynamic and metabolic changes. For hemodynamics and oxCCO monitoring, the optimal wavelengths aim to reduce the quantification errors compared with that obtained with broadband spectra between 780 and 900 nm. In this study, we also proposed to quantify the changes of the cytochrome b and c. We can see in Fig. 11 that the addition of the cytochromes b and c in the modified Beer–Lambert system helps to better resolve oxCCO changes compared with the model based on three chromophores (${\mathrm{HbO}}_{2}$, Hb, and oxCCO), see Fig. 10. Indeed, we can see that the quantification errors in oxCCO are lower when all cytochromes are considered, see Table 3. This result is interesting as it may help to obtain robust devices to monitor brain metabolism, which could be a hyperspectral device for intraoperative brain mapping or even a NIRS device for bedside monitoring. The monitoring of the oxidation state of CCO could help to obtain a more direct biomarker of neuronal activity, to detect brain injuries,^16^^,^^57^ and to better understand the neurovascular coupling.^58^

The optimization procedure does not help to resolve changes in cytochrome b and c in a significant way. Indeed, we can see in Figs. 11 and 12 that oxCytb and oxCytc measurements are really noisy. A two-sample T-test tells us that changes measured at the level of the activated grey matter are not significantly different from those measured on non-activated grey matter ($p_{\text{values}}$ are mainly higher than 0.1). This is maybe due to the noise addition, coupled with the fact that the attenuation changes due to the oxidized cytochrome b and c are rather low compared with those of hemoglobin and oxCCO.

The optimization procedure helps to obtain smaller quantification errors compared with the literature configurations. For hemodynamic monitoring, increasing the number of wavelengths from 2 to 10 helps to reduce linearly the quantification error from 8% to 26%, see Fig. 9. However for hemodynamic and metabolic monitoring, we do not observe a significant decrease in the quantification error compared with Bale’s spectral configuration if using 6, 8, or 10 optimal wavelengths (quantification errors of 32%, 33%, and 33%, respectively, see Fig. 10). Thus, the number of wavelengths of a hyperspectral camera could be reduced to six to resolve hemodynamic and CCO changes. It could help to optimize several critical parameters for intraoperative functional brain mapping applications such as the signal-to-noise ratio. However, the availability of light sources or spectral filters at the identified wavelengths may limit the development of the hyperspectral camera. To address this limitation, we plan to restrict the optimization procedure to a number of wavelengths selected from the available light sources or spectral filters. For each wavelength, the spectral bandwidth can also be incorporated into the procedure. As proposed by Leadley et al,^32^ we will also investigate in a future study the influence of extinction coefficient uncertainties on chromophore quantification.

We also showed that a separate quantification of hemodynamics and oxCCO leads to a better estimation of the chromophore changes, see Table 3. The optimization procedure helps to identify the best wavelength combination in the range 400 to 1000 nm, which reduces the quantification errors in ${\mathrm{HbO}}_{2}$, Hb, and oxCCO by 47%, 57%, and 57% compared with the gold standard of 121 wavelengths between 780 and 900 nm. The configuration of six wavelengths is 468, 482, 610, 753, 814, and 907 nm to monitor ${\mathrm{HbO}}_{2}$ and Hb changes and 608, 650, 661, 672, 820, and 865 nm to monitor oxCCO changes. The separate quantification method should be taken with caution because it distorts the link between chromophores. Moreover, this could lead to incorrect interpretations between the chromophore changes and the physiological status of the patient. However, this method could be interesting for some clinical applications. For example, the method can be used to identify a spectral configuration for precise monitoring of Hb changes with the idea to define a blood oxygen level dependent-like contrast.^58^ In intraoperative brain mapping studies, this approach has been proposed by several research groups. The authors used a single illumination at 605^59^ or 610 nm^60^ combined with a monochrome camera to assess Hb absorption. For these two wavelengths, the authors considered that the signal changes were mainly due to Hb because the absorption related to ${\mathrm{HbO}}_{2}$ is negligible compared with that of Hb (the ratio between Hb molar extinction coefficient to that of ${\mathrm{HbO}}_{2}$ is ≈6.26). Although the contribution of ${\mathrm{HbO}}_{2}$ is small compared with that of Hb, the signal measured with this type of device cannot accurately measure variations in Hb concentration. Our method solves this problem.

## 5 Conclusion

In this paper, we proposed two main contributions. First, we presented a digital instrument simulator to optimize the development of hyperspectral systems for intraoperative brain mapping studies. This simulator is based on a realistic digital phantom of an exposed cortex computed with white Monte Carlo simulations. A C++ software was developed to reconstruct temporal perturbations of the absorption coefficient using only one simulation. Second, we presented an optimization procedure based on the genetic algorithm to identify the best wavelength combinations in the visible and near-infrared range to quantify changes in ${\mathrm{HbO}}_{2}$, Hb, oxCCO, oxCytb, and oxCytc. With the digital instrument simulator, we can improve the accuracy of actual preclinical and clinical optical devices used in brain mapping applications with the consideration of the impact of the large blood vessels on the path length. We also proposed several spectral configurations to monitor hemodynamic and metabolic changes in grey matter that aimed to reduce the quantification error of changes in ${\mathrm{HbO}}_{2}$, Hb, and oxCCO compared with the configuration proposed in the literature. This digital instrument simulator and this optimization framework could be used to optimize the design of hyperspectral imaging devices,^61^^,^^62^ with the objective of transforming current practice toward an all-optical, real-time, quantitative, and accurate imaging approach, which could significantly help neurosurgeons, enhance the efficacy of the treatment, and ultimately improve the quality of life and functional outcomes of the patients after the brain tumor resection.

## Supplementary Material



## Data Availability

In support of open science, the code presented in this article is publicly available on https://github.com/CCaredda/White-Monte-Carlo/.
