# Supplementary material for: Digital instrument simulator to optimize the development of hyperspectral systems: application for intraoperative functional brain mapping
Source: J Biomed Opt. 2024 Dec 2;30(2):023513. doi: 10.1117/1.JBO.30.2.023513 (PMC11610766; doi:10.1117/1.JBO.30.2.023513)
Supplement: Supplementary file 1 [file JBO_030_023513_SD001.pdf]

A digital instrument simulator to optimize the development of hyperspectral systems: application for intraoperative functional brain mapping

Charly Caredda[a*], Frédéric Lange[b], Luca Giannoni[c], Ivan Ezhov[d], Thiébaud Picart[e], Jacques Guyotat[e], Ilias Tachtsidis[b], Bruno Montcel[a]
[a]Univ Lyon, INSA-Lyon, Université Claude Bernard Lyon 1, UJM-Saint Etienne, CNRS, Inserm, CREATIS UMR 5220, U1294, F69100, Lyon, France
[b]Department of Medical Physics and Biomedical Engineering, University College London, UK
[c]Department of Physics and Astronomy, University of Florence, Italy
[d]Technical University of Munich, Germany
[e]Service de Neurochirurgie D, Hospices Civils de Lyon, Bron, France

*****Charly Caredda, charly.caredda@creatis.insa-lyon.fr

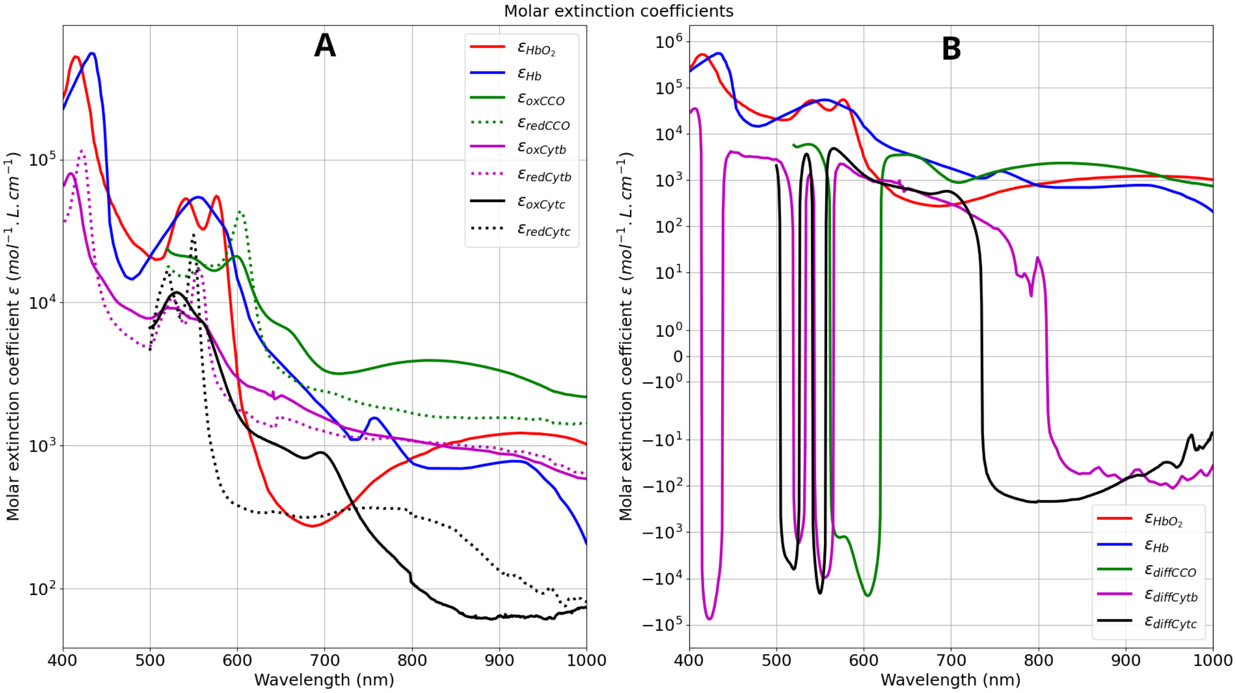

Fig 1 Molar extinction spectra of oxygenated, deoxygenated hemoglobin (HbO_2 and Hb), cytochromes b, c and cytochrome-c-oxidase ($Cytb$, $Cytc$, CCO). A - Molar extinction spectra of the reduced (red) and oxidized (ox) states of cytochromes. B - Difference molar extinction molar between the oxidized and reduced states of cytochromes.

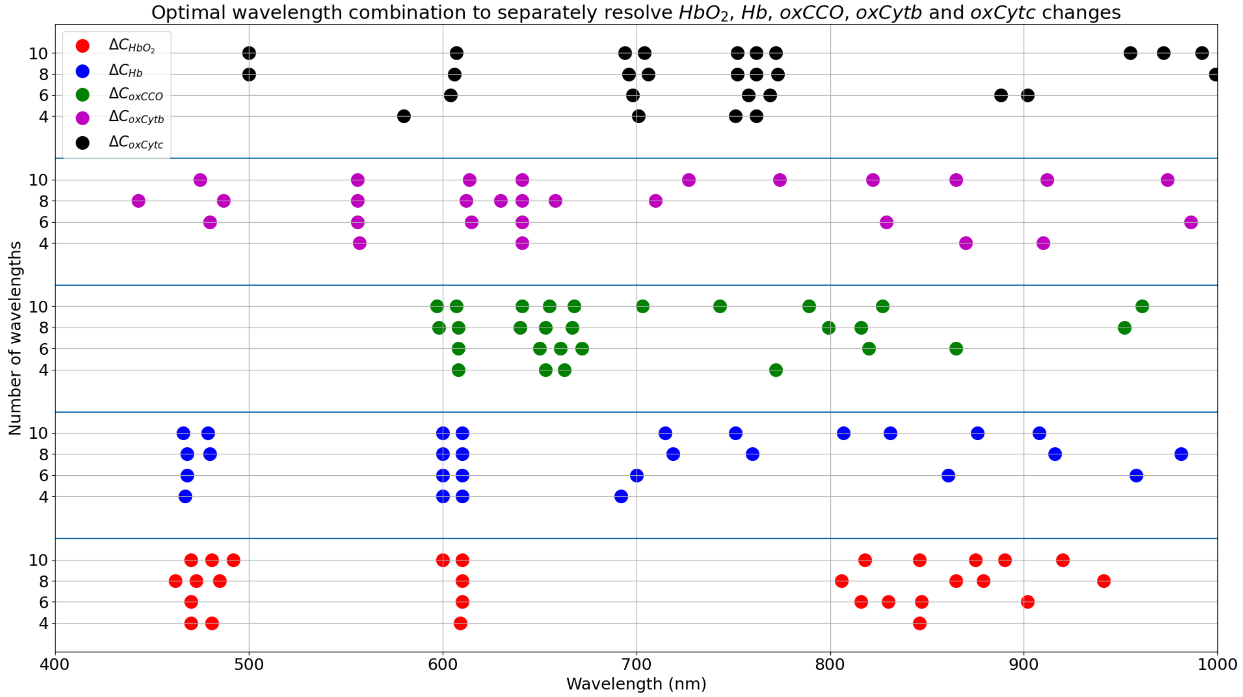

Fig 2 Optimal combinations of 4, 6, 8 and 10 wavelengths to separately resolve changes in C_{HbO_2}, C_{Hb}, C_{oxCCO}, C_{oxCytb} and C_{oxCytc}.
